# SUPPORT Tools for evidence-informed health Policymaking (STP) 13: Preparing and using policy briefs to support evidence-informed policymaking

**DOI:** 10.1186/1478-4505-7-S1-S13

**Published:** 2009-12-16

**Authors:** John N Lavis, Govin Permanand, Andrew D Oxman, Simon Lewin, Atle Fretheim

**Affiliations:** 1 Centre for Health Economics and Policy Analysis, Department of Clinical Epidemiology and Biostatistics, and Department of Political Science, McMaster University, 1200 Main St. West, HSC-2D3, Hamilton, ON, Canada, L8N 3Z5; 2 Health Evidence Network, World Health Organization Regional Office for Europe, Scherfigsvej 8, Copenhagen, Denmark DK-2100; 3 Norwegian Knowledge Centre for the Health Services, P.O. Box 7004, St. Olavs plass, N0130 Oslo, Norway; 4 Norwegian Knowledge Centre for the Health Services, P.O. Box 7004, St. Olavs plass, N0130 Oslo, Norway; Health Systems Research Unit, Medical Research Council of South Africa; 5 Norwegian Knowledge Centre for the Health Services, P.O. Box 7004, St. Olavs plass, N0130 Oslo, Norway; Section for International Health, Institute of General Practice and Community Medicine, Faculty of Medicine, University of Oslo, Norway

## Abstract

*This article is part of a series written for people responsible for making decisions about health policies and programmes and for those who support these decision makers*.

Policy briefs are a relatively new approach to packaging research evidence for policymakers. The first step in a policy brief is to prioritise a policy issue. Once an issue is prioritised, the focus then turns to mobilising the full range of research evidence relevant to the various features of the issue. Drawing on available systematic reviews makes the process of mobilising evidence feasible in a way that would not otherwise be possible if individual relevant studies had to be identified and synthesised for every feature of the issue under consideration. In this article, we suggest questions that can be used to guide those preparing and using policy briefs to support evidence-informed policymaking. These are: 1. Does the policy brief address a high-priority issue and describe the relevant context of the issue being addressed? 2. Does the policy brief describe the problem, costs and consequences of options to address the problem, and the key implementation considerations? 3. Does the policy brief employ systematic and transparent methods to identify, select, and assess synthesised research evidence? 4. Does the policy brief take quality, local applicability, and equity considerations into account when discussing the synthesised research evidence? 5. Does the policy brief employ a graded-entry format? 6. Was the policy brief reviewed for both scientific quality and system relevance?

## About STP

*This article is part of a series written for people responsible for making decisions about health policies and programmes and for those who support these decision makers. The series is intended to help such people ensure that their decisions are well-informed by the best available research evidence. The SUPPORT tools and the ways in which they can be used are described in more detail in the Introduction to this series *[1]. *A glossary for the entire series is attached to each article (see Additional File *1*). Links to Spanish, Portuguese, French and Chinese translations of this series can be found on the SUPPORT website http://www.support-collaboration.org. Feedback about how to improve the tools in this series is welcome and should be sent to: STP@nokc.no*.

## Scenarios

*Scenario 1: You are a senior civil servant and have been sent a policy brief that describes the research evidence about an issue that is of growing concern to the Minister. You are responsible for ensuring that the policy brief profiles research evidence in a way that informs different elements of the issue and recognises the importance of drawing on both local and global evidence. You want to ensure that the policy brief won't place the Minister in an awkward position by making a recommendation that is not politically or economically feasible*.

*Scenario 2: You work in the Ministry of Health and have been given a few hours to prepare an assessment of a policy brief that has been sent to the Ministry on a high-priority issue. All that you have been told is that this policy brief is different in a number of ways to the type of policy brief that you have produced in the past including the way in which it profiles research evidence about a problem, the options and implementation considerations, and the fact that it does not conclude with a specific recommendation*.

*Scenario 3: You work in an independent unit that supports the Ministry of Health in its use of research evidence in policymaking. You are preparing a policy brief for both the Ministry and key stakeholders to profile what is known and not known about a problem, options for addressing it, and implementation considerations. You have been told to prepare the brief in a systematic way and to report the methods and findings in a transparent and readily understandable way, but you want guidance on how to be both thorough and efficient in your work*.

## Background

For policymakers (Scenario 1), this article suggests a number of questions that they might ask themselves or their staff to consider when assessing a policy brief. For those who support policymakers (Scenarios 2 and 3), this article suggests a number of questions to guide the assessment of a policy brief or the preparation of one.

Three major shifts have occurred recently in the focus of many efforts to package research evidence for policymakers. Firstly, there has been a shift from packaging single studies to packaging systematic reviews of studies that address typical policy-relevant questions. A number of research groups, including the SUPPORT collaboration http://www.support-collaboration.org/, now produce policymaker-friendly summaries of systematic reviews. These summaries always highlight the key messages from the review but some of them, like SUPPORT summaries, also address considerations related to quality, local applicability, and equity [2]. This shift has made it easier for policymakers to scan broadly across large bodies of research evidence. And it has also enabled them to extract what they need to know easily from particular systematic reviews that directly address key features of any policy issue of interest.

Secondly, there have been more recent complementary efforts to package systematic reviews (together with local research evidence) in the form of a new product - the policy brief - which mobilises the best available research evidence on high-priority issues [3]. For policy briefs, the starting point is the issue and *not *the related research evidence that has been produced or identified. Once an issue is prioritised, the focus then turns to mobilising the full range of research evidence addressing the different features of the issue concerned. These include the underlying problem, options to address the problem, and key implementation considerations. Drawing on available systematic reviews makes the process of evidence mobilisation feasible in a way that would not otherwise be possible if single studies had to be identified and synthesised for all the features of the issue. In this article, we have restricted our use of the term 'policy brief' to those products matching this description exactly. But the term has also been applied elsewhere to many other types of products prepared by those supporting policymakers. The appropriation of this term by those involved in producing and supporting the use of research evidence reflects perhaps their increasing orientation to the needs and contexts of policymakers.

Evidence-packaging mechanisms and policy briefs in particular have been developed largely as a response to the findings of systematic reviews of factors influencing the use of research evidence in policymaking [4,5]. Three factors in particular have emerged as significant. These are: 1. Timing or timeliness, 2. Accordance between the research evidence and the beliefs, values, interests, or political goals and strategies of policymakers and stakeholders, and 3. Interactions between researchers and policymakers.

Having access to both a stock of the summaries of systematic reviews and policy briefs helps to address the need that policymakers have for timely inputs to policymaking processes [6]. Review summaries and policy briefs can typically be produced in days and weeks rather than the months or years required to prepare a systematic review from scratch. Undertaking primary research (i.e. original studies) can be similarly and often more time intensive. Evidence-packaging mechanisms, and policy briefs in particular, can also make it easier for policymakers and other stakeholders to determine whether and how the available research evidence accords with their own beliefs, values, interests, or political goals and strategies. With a problem clarified, what is known and not known about the options clearly described, and key implementation considerations clearly flagged, policymakers may be more readily able to identify viable ways forward.

Thirdly, changes have occurred in the purpose for which packaged research evidence has typically been produced. Policy briefs are increasingly used as an input into policy dialogues involving individuals drawn from those who will be involved in, or affected by, decisions about a particular issue. These dialogues provide the opportunity for greater interaction between researchers and policymakers. Dialogues in which research evidence is just one input in a policy discussion form the focus of Article 14 in this series [7].

The formats used for evidence-packaging have often been developed in response to the few available empirical studies of the preferences of health policymakers for different kinds of mechanisms (and *not *their usage or effects, which typically have not been evaluated) [4,8]. These studies have revealed a need amongst policymakers to have formats that both provide graded entry to the full details of a review *and *facilitate assessment of decision-relevant information [4]. A graded-entry format of one page of take-home messages, a three-page executive summary that summarises the full report, and a 25-page report (i.e. a 1:3:25 format) has shown to be particularly promising [9]. Presumably, either the one- or three-page summary should follow a structured format [10]. Structured abstracts have been found to have an effect on intermediate outcomes such as searchability, readability and recall among healthcare providers. However, no studies have compared full text to structured abstracts and no studies have examined the impact of format features on policymakers [11]. Decision-relevant information can include the important impacts (both benefits and harms) and costs (i.e. resources used) of policy and programme options, as well as local applicability and equity considerations [4].

## Questions to consider

The following questions can be used to guide the preparation and use of policy briefs to support evidence-informed policymaking:

1. Does the policy brief address a high-priority issue and describe the relevant context of the issue being addressed?

2. Does the policy brief describe the problem, costs and consequences of options to address the problem, and the key implementation considerations?

3. Does the policy brief employ systematic and transparent methods to identify, select, and assess synthesised research evidence?

4. Does the policy brief take quality, local applicability, and equity considerations into account when discussing the research evidence?

5. Does the policy brief employ a graded-entry format?

6. Was the policy brief reviewed for both scientific quality and system relevance?

### 1. Does the policy brief address a high-priority issue and describe the relevant context of the issue being addressed?

Policy briefs are distinguished most clearly from other packaged evidence summaries by the fact that they begin with the explicit identification of a high-priority issue. In instances where an issue has been on the agenda of key stakeholders for some time, policy briefs may act as a way to spur progress. This is highlighted in the example shown in Table 1 of low coverage rates for artemisinin-based combination therapies (ACT) to treat uncomplicated falciparum malaria in sub-Saharan African countries. Alternatively, if the issue is relatively new, the policy brief may play an agenda-setting role. Either way, it is critical that the issue is deemed a priority by at least some key stakeholders. Ideally the prioritisation process should also be systematic and transparent and Article 3 in this series outlines an approach for achieving this [12].

**Table 1:** Outline of a policy brief about supporting the widespread use of a new, highly effective treatment for malaria in an African country

What problem has been identified?
• The overarching problem is one of low coverage rates for artemisinin-based combination therapies (ACT) to treat uncomplicated falciparum malaria in sub-Saharan Africa. Key features of the problem include:
• A high incidence of, and death rates from, malaria
• Existing treatments have much lower cure rates than ACT. However, patients often favour existing treatments because of their past experiences and the higher price of ACT
• The national malaria control policy, treatment guidelines, and drug formulary in many countries do not all support the prescription, dispensing and use of ACT
• Delivery arrangements for ACT often rely primarily on physicians but not everyone has regular access to them and many are comfortable receiving care from community health workers. Financial arrangements favour existing treatments over ACT (which is much more expensive) yet some patients are sceptical about heavily subsidised medication. Governance arrangements often do not allow community health workers to prescribe ACT and do not protect against counterfeit or substandard drugs
What information do systematic reviews provide about three viable options to address the problem?
• Each of the following three options was assessed in terms of the likely benefits, harms, costs (and cost-effectiveness), key elements of the policy option if it was tried elsewhere, and the views and experiences of relevant stakeholders:
• Enlarge the scope of practice for community health workers to include the diagnosis of malaria and prescription of ACT (governance arrangements), introduce target payments for achieving a defined coverage rate for ACT treatment (financial arrangements), and provide them with training and supervision for the use of both rapid diagnostic tests and prescribing (delivery arrangements)
• Introduce partial subsidies for both rapid diagnostic tests and ACT within the private sector where much care is provided in urban areas (financial arrangements)
• Restrict the types of anti-malaria drugs that can be imported and introduce penalties for those found dispensing counterfeit or substandard drugs (governance arrangements) and make changes to the national malaria control policy and drug formulary to ensure that ACT is the recommended first-line treatment
• Important uncertainties about each option's benefits and potential harms were flagged in order to give them particular attention as part of any monitoring and evaluation plan put into place
What key implementation considerations need to be borne in mind?
• A number of barriers to implementation were identified, among which were the familiarity of some patients and healthcare providers with existing treatment options and their resistance to change. Systematic reviews about the effects of mass media campaigns, the effects of strategies for changing healthcare provider behaviour generally, *and *for influencing prescribing and dispensing specifically, all proved helpful in deciding how to address these barriers
Notes about the supporting evidence base:
• Six systematic reviews about anti-malarial drugs had been published since the release of the World Health Organization guidelines in 2006, all of which lent further support to ACT as the recommended first-line treatment
• Of the systematic reviews identified: two addressed relevant governance arrangements, six addressed financial arrangements, five addressed specific configurations of human resources for health, and fifteen addressed implementation strategies, many of which could be supplemented by local studies

A second key feature of policy briefs is that they are typically *context-specific*. Describing the key features of a context in the policy brief is important as a way of creating a level playing field among policy brief readers. Table 2 highlights issues related to limited or inequitable access to sustainable, high-quality community-based primary healthcare in Canada. There, as the policy brief explained, the issue could only be understood in the context of the particular features of Canadian primary healthcare and the existence of 'private delivery/public payment' arrangements with physicians. These are of particular importance in this context for they have meant historically that most primary healthcare in Canada is delivered by physicians working in private practice with first-dollar, public (typically fee-for-service) payment [13]. Improving access in creative ways, including the use of collaborative practice models, requires an understanding that: 1. Physicians tend to be wary of potential infringements on their professional and commercial autonomy, 2. No other healthcare providers at this time can secure the public payment required to function independently as primary healthcare providers on a viable scale, and 3. Many forms of care (including prescription drugs and home care services) would still not be covered [14].

**Table 2:** Outline of a policy brief about improving access to high quality primary healthcare in Canada

What problem has been identified?
• The problem is limited or inequitable access to sustainable, high-quality community-based primary healthcare in federal, provincial, and territorial publicly-funded health systems in Canada. Key characteristics of the problem include:
• Chronic diseases represent a significant share of the common conditions that must be prevented or treated by the primary healthcare system
• Access to cost-effective programmes, services and drugs in Canada is not ideal. This is the case both when Canadians identify their own care needs or (more proactively on the part of healthcare providers) when they have an indication (or need) for prevention or treatment, particularly for chronic disease prevention and treatment
• Health system arrangements have not always supported the provision of cost-effective programmes, services and drugs. Many Canadians do not:
1. Have a regular physician or place of care
2. Receive effective chronic-disease management services, or
3. Receive care in a primary healthcare practice that uses an electronic health record, faces any financial incentive for quality, or provides nursing services
What is more difficult to determine is the proportion of physicians who receive effective continuing professional development for chronic disease management and the proportion of primary healthcare practices that:
1. Are periodically audited for their performance in chronic disease management
2. Employ physician-led or collaborative practice models, and
3. Adhere to a holistic primary healthcare model's (the Chronic Care Model's) key features [21]
What information do systematic reviews provide about three viable options to address the problem?
• Each of the following three options was assessed in terms of its likely benefits, harms, costs (and cost-effectiveness), its key elements if it had been tried elsewhere, and stakeholder views about and experiences with it:
• Support the expansion of chronic disease management in physician-led care through a combination of electronic health records, target payments, continuing professional development, and auditing of their primary healthcare practices
• Support the targeted expansion of inter-professional, collaborative practice primary healthcare
• Support the use of the Chronic Care Model in primary healthcare settings. This model entails the combination of self-management support, decision support, delivery system design, clinical information systems, health system, and community
• Important uncertainties about each option's benefits and potential harms were flagged. This was done in order to give these issues particular attention within any monitoring and evaluation plan put into place
What key implementation considerations need to be borne in mind?
• Little empirical research evidence could be identified about implementation barriers and strategies. Four of the implementation barriers identified were:
1. Initial wariness amongst some patients of potential disruptions to their relationship with their primary healthcare physician
2. Wariness on the part of physicians (particularly older physicians) of potential infringements on their professional and commercial autonomy
3. The organisational scale required for some of the options is not viable in many rural and remote communities, and
4. Hesitancy on the part of governments about broadening the breadth and depth of public payment for primary healthcare, particularly during a recession
Notes about the supporting evidence base:
• Dozens of relevant systematic reviews were identified, some of which addressed an option directly and others of which addressed elements of one or more options [14]

### 2. Does the policy brief describe the problem, costs and consequences of options to address the problem, and the key implementation considerations?

A policy brief would ideally describe different features of a problem, what is known (and not known) about the costs and consequences of options for addressing the problem, and key implementation considerations. As outlined in Article 4, a problem can be understood in one or more of the following terms [15]:

1. The nature and burden of the actual common diseases and injuries that the healthcare system must prevent or treat

2. The cost-effective programmes, services and drugs that are needed for prevention and treatment, and

3. The broader health system arrangements that determine access to, and the use of, cost-effective programmes, services and drugs, including how they affect particular groups.

A policy brief would help to clarify the problem by diagnosing it in one or more of these terms.

Ideally, the number of options described in a brief that is to be presented to senior policymakers would conform to local document conventions. Three-option models, for instance, are familiar to many policymakers. But regardless of the number selected, each option in the policy brief can be characterised in terms of:

• The benefits of each option

• The harms of each option

• The costs of each option or their relative cost-effectiveness (if possible)

• The degree of uncertainty related to these costs and consequences (so that monitoring and evaluation can focus on particular areas of uncertainty if any given option is pursued)

• Key elements of the policy option if it has been tried elsewhere and adaptation is being considered, and

• Stakeholder views about and experiences with each option

A policy brief would help to make clear the trade-offs involved in selecting one option over others. If the options are not designed to be mutually exclusive, a policy brief would also help to make clear the benefits of combining particular elements of the different options and which *combination *of options might bring about positive synergies. Alternatively, the elements from one or more individual options could be presented first, followed by 'bundles' of options combining different elements in various ways.

Barriers to implementation (outlined in further detail in Article 6 in this series) are located at different levels, ranging from the consumer (citizen or healthcare recipient) level through to healthcare providers, organisations, and broader systems [16]. Policy briefs would help to identify these barriers and describe what can reasonably be expected (again, in terms of benefits, harms, and costs) as a result of pursuing alternative implementation strategies to address these barriers. A policy brief could also identify considerations related to the preparation of a monitoring and evaluation plan. Table 3 provides a possible outline for a policy brief.

**Table 3:** Possible outline of a policy brief

**Title **(possibly in the form of a compelling question)			
**Key messages **(possibly as bullet points)			
• What is the problem?			
• What do we know (and not know) about viable options to address the problem?			
• What implementation considerations need to be borne in mind?			
Report			
• Introduction that describes the issue and the context in which it will be addressed			
• Definition of the problem such that its features can be understood in one or more of the following terms:			
1. The nature and burden of common diseases and injuries that the healthcare system must prevent or treat			
2. The cost-effective programmes, services and drugs that are needed for prevention and treatment, and			
3. The health system arrangements that determine access to and use of cost-effective programmes, services and drugs, including how they affect particular groups			
• Options for addressing the problem, with each one assessed in a table (an example is shown below)			
			

**Category of finding**		**Nature of findings from systematic reviews and other available research evidence**	

Benefits			
Harms			
Costs and cost-effectiveness			
Uncertainty regarding benefits and potential harms			
Key elements of the option (how and why it works)			
Stakeholders' views and experiences			

• Implementation considerations, with potential barriers to implementing the options assessed in a table (please see example below), each viable implementation strategy also assessed in table (please see example above), and suggestions for a monitoring and evaluation plan			

			

**Levels**	**Option 1**	**Option 2**	**Option 3**

Consumer			
Healthcare provider			
Organisation			
System			
**Additional content that could appear on a cover page or in an appendix:**			
• A list of authors and their affiliations			
• A list of those involved in establishing the terms of reference for the policy brief and their affiliations			
• A list of key informants who were contacted to gain additional perspectives on the issue and to identify relevant data and research evidence, and their affiliations			
• A list of funders (for the organisation producing the policy brief and for the policy brief itself)			
• A statement about conflicts of interest among authors			
**Additional content that could appear in boxes or in an appendix**			
• Methods used to identify, select, and assess synthesised research evidence (including assessments of quality, local applicability and equity considerations)			
• Review process used to ensure the scientific quality and system relevance of the policy brief			

### 3. Does the policy brief employ systematic and transparent methods to identify, select, and assess synthesised research evidence?

Policymakers and a wide range of stakeholders who will be involved in or affected by a decision, are the main audience of a policy brief. Research language should therefore be kept to a minimum as most people will be unfamiliar with it. A policy brief, nevertheless, should still ideally describe how synthesised research evidence was identified, selected and assessed in ways that are easily understood. This objective can be achieved by using techniques such as explanatory 'boxes' within the brief to clarify or highlight particular concepts, or through the inclusion of additional appendices. The methods, too, should be systematic in nature and reported in a transparent yet understandable way. For example, users could be provided with a description of how systematic reviews addressing the benefits and harms of particular health system arrangements were identified through a search of continuously updated databases containing reviews in particular domains. This could provide significant reassurance to readers that most, if not all, key reviews had been found and that few, if any, key reviews had been missed.

### 4. Does the policy brief take quality, local applicability, and equity considerations into account when discussing the research evidence?

Systematic reviews may be of high or low quality, their findings may be highly applicable to a given policymaker's setting or of very limited applicability, and they may or may not give consideration to the impacts an option is likely to have on disadvantaged groups, and on equity in a specific setting. Ideally, a policy brief would flag such variations for policymakers and other readers. As outlined in Article 8, explicit criteria are available to assist with quality assessments [17]. Importantly, some databases of systematic reviews, such as Rx for Change http://www.rxforchange.ca, provide quality ratings for all reviews contained in the database. If possible, a policy brief would provide a quality review for all systematic reviews from which key messages have been extracted. Explicit criteria are also available to assist with local applicability assessments and these are outlined in further detail in Article 9 [18]. Given that policy briefs are typically context-specific, a policy brief would also ideally comment on the local applicability of the findings of any systematic reviews that are critical to an understanding of the impacts of any options being considered. Equity considerations can also be addressed using explicit criteria (see Article 10) [19]. A policy brief should also note in its introduction whether any groups have been given particular attention in the brief. Group-specific key messages could be added to the overall key messages in each section.

### 5. Does the policy brief employ a graded-entry format?

A policy brief would ideally allow busy policymakers and other readers to scan the key messages quickly in order to determine whether these corresponded sufficiently closely to their key issue of concern and context to warrant reading the entire document. A graded-entry format could take a number of forms. These could be achieved, for example, through a 1:3:25 format - i.e. *one *page of take-home messages, a *three*-page executive summary, and a *25*page report [9]. Or a brief may take the form of a 1:12 format, with one page of take-home messages followed by a 12-page report. Whatever form is chosen, the minimum that a policy brief should contain is a list of key messages, a report, and a reference list for those who wish to read more. The key messages would range from the identification of the problem through what is known about the options, and the key considerations for implementation.

A number of other features of a policy brief could engage potential readers and facilitate assessments of who was involved in preparing, informing and funding it. The title of a policy brief could be worded in a way that would engage policymakers and other stakeholders. This could be achieved, for example, by using a compelling question as a title. The cover and/or the acknowledgements section of a policy brief could provide a list of authors and their affiliations. It could also include a list of those involved in establishing the terms of reference of the policy brief, a list of the key informants contacted for additional perspectives on the issue and to identify relevant data and research evidence, and their affiliations. A list of funders for both the organisation producing the policy brief and the policy brief itself, and a statement about any conflicts of interest among authors could also form part of the policy brief document.

### 6. Was the policy brief reviewed for both scientific quality and system relevance?

Policy briefs need to meet two standards: scientific quality and system relevance. To ensure this, the review process could involve at least one policymaker, at least one other stakeholder, and at least one researcher. This so-called *merit review *process differs from a typical *peer review *process that would typically only involve researchers in the review process, and hence focus primarily on scientific quality. Involving policymakers and other stakeholders can help to ensure the brief's relevance to the health system.

## Conclusion

Policy briefs are a new approach to supporting evidence-informed policymaking. Their preparation and use continues to evolve through practical experience. Evaluations of this new approach are needed in order to improve our understanding of which particular design features are well received for particular types of issues and in particular contexts. Describing the different features of a problem may, for example, be perceived as being particularly important for highly politicised topics where the very nature of the problem is contentious. Taking equity considerations into account through a focus on only one group may be perceived as inappropriate in political systems that may have a long tradition of either addressing all major ethnocultural groups in policy documents or perhaps of focusing on no groups in particular. Evaluations are also necessary as a way of improving our understanding of whether, and how, policy briefs influence policymaking. Table 4 provides a description of one approach to the formative evaluation of policy briefs.

**Table 4:** An example of an approach to the formative evaluation of a policy briefs series

• The McMaster Health Forum surveys those to whom it sends a policy brief, with the long term goal of identifying which design features work best for particular types of issues, and in which particular health system contexts. Participation is voluntary, confidentiality assured, and anonymity safeguarded
• Twelve features of the policy briefs series are the focus of questions in the formative evaluation survey:
• Describes the context of the issue being addressed
• Describes different features of the problem, including (where possible) how it affects particular groups
• Describes three options for addressing the problem
• Describes key implementation considerations
• Employs systematic and transparent methods to identify, select, and assess synthesised research evidence
• Takes quality considerations into account when discussing the research evidence
• Takes local applicability considerations into account when discussing the research evidence
• Takes equity considerations into account when discussing the research evidence
• Does not conclude with particular recommendations
• Employs a graded-entry format (i.e. a list of key messages and a full report)
• Includes a reference list for those who want to read more about a particular systematic review or research study, and
• Is subject to a review by at least one policymaker, at least one stakeholder, and at least one researcher. This process is termed a *merit *review to distinguish it from a standard *peer *review which would typically only involve researchers in the review process
• For each design feature, the survey asks:
• How useful did they find this approach (on a scale from 1 = Worthless to 7 = Useful)?
• Are there any additional comments or suggestions for improvement?
• The survey also asks:
• How well did the policy brief achieve its purpose, namely to present the available research evidence on a high-priority issue in order to inform a policy dialogue where research evidence would be just one input to the discussion (on a scale from 1 = Failed to 7 = Achieved)?
• What features of the policy brief should be retained in future?
• What features of the policy brief should be changed in future?
• What key stakeholders can do better or differently to address the high-priority issue and what they personally can do better or differently?
• Their role and background (so that the McMaster Health Forum can determine if different groups have different views and experiences related to policy briefs)
• The Evidence-Informed Policy Networks (EVIPNet) operating in Africa, Asia and the Americas plan to use a similar approach in the formative evaluation of their policy briefs

## Resources

### Useful documents and further reading

- Research Matters. Knowledge Translation: A 'Research Matters' Toolkit. Ottawa, Canada: International Development Research Centre: http://www.idrc.ca/research-matters/ev-128908-201-1-DO_TOPIC.html - Source of additional examples of policy briefs (Chapter 8) and, most importantly, guidance about effective communication (Chapters 6 and 7)

- Canadian Health Services Research Foundation. Communication Notes: Reader-Friendly Writing - 1:3:25. Ottawa, Canada: Canadian Health Services Research Foundation: http://www.chsrf.ca/knowledge_transfer/pdf/cn-1325_e.pdf - Source of advice about writing for an audience of policymakers and other stakeholders

- Lavis JN, Boyko JA: *Evidence Brief: Improving Access to Primary Healthcare in Canada*. Hamilton, Canada: McMaster Health Forum; 2009 [14] - Example of a policy brief for a specific country (Canada)

- Oxman AD, Bjorndal A, Flottorp SA, Lewin S, Lindahl AK: *Integrated Health Care for People with Chronic Conditions*. Oslo, Norway: Norwegian Knowledge Centre for the Health Services; 2008 [20]: http://www.kunnskapssenteret.no/Publikasjoner/5114.cms?threepage=1 - Example of a policy brief that provides an exhaustive review of the potential elements of policy options before bundling them together into three viable options for a specific country (Norway)

### Links to websites

- Health Evidence Network/European Observatory on Health Systems and Policies: http://www.euro.who.int/hen/policybriefs/20070327_1 - Source of policy briefs targeted at policymakers in the World Health Organization's European Region

- Program in Policy Decision-Making (PPD)/Canadian Cochrane Network and Centre (CCNC) database: http://www.researchtopolicy.ca/search/reviews.aspx - Source of policy briefs as well as systematic reviews and overviews of systematic reviews (with links to policymaker-friendly summaries of systematic reviews and overviews of systematic reviews)

- SUPPORT Collaboration: http://www.support-collaboration.org - Example of a source of policymaker-friendly summaries of systematic reviews relevant to low- and middleincome countries

## Competing interests

The authors declare that they have no competing interests.

## Authors' contributions

JNL prepared the first draft of this article. GP, ADO, SL and AF contributed to drafting and revising it.

## Acknowledegements

Please see the Introduction to this series for acknowledgements of funders and contributors. In addition, we would like to acknowledge Sandy Campbell and staff in the Ontario Ministry of Health and Long-Term Care (MoHLTC) Planning Unit for helpful comments on an earlier version of this Article.

This article has been published as part of *Health Research Policy and Systems *Volume 7 Supplement 1, 2009: SUPPORT Tools for evidence-informed health Policymaking (STP). The full contents of the supplement are available online at http://www.health-policy-systems.com/content/7/S1.

## Supplementary Material

Additional file 1 Glossary Click here for file
